# Editorial: Community series in epigenetics of the immune component of inflammation-volume II

**DOI:** 10.3389/fimmu.2023.1266133

**Published:** 2023-08-15

**Authors:** Yan-Jun Liu, Hai-Jing Zhong, Haitao Wang, Cheong-Meng Chong, Guan-Jun Yang

**Affiliations:** ^1^ State Key Laboratory for Managing Biotic and Chemical Threats to the Quality and Safety of Agro-products, Ningbo University, Ningbo, Zhejiang, China; ^2^ Laboratory of Biochemistry and Molecular Biology, School of Marine Sciences, Ningbo University, Ningbo, China; ^3^ College of Pharmacy, Jinan University, Guangzhou, Guangdong, China; ^4^ Center for Cancer Research, National Cancer Institute (NIH), Bethesda, MD, United States; ^5^ State Key Laboratory of Quality Research in Chinese Medicine, Institute of Chinese Medical Sciences, University of Macau, Macao, Macao SAR, China

**Keywords:** epigenetics, immune response, inflammation, genome modification, posttranscriptional modifications, post-translational modifications

Epigenetics is also known as pseudogenetics or postgenetics, and explores heritable changes in gene expression or cell phenotype through certain mechanisms, without changes in the DNA sequence in biology and specific genetics (1, 2). Inflammation is a basic pathological process that occurs in living tissues with a vascular system in response to the stimulation of various damage factors (3–6). It is well-known that inflammation is mediated by a variety of immune components (including complements, cytokines, chemokines, transcriptional factors, pattern recognition receptors, etc.) secreted or expressed by immune/non-immune cells (2). Mounting evidence supports that epigenetic modifications are associated with the occurrence, development, and resolution of inflammation via remodeling immune/non-immune cells and the microenvironment (5, 6), thus promoting or repressing the progression of many inflammatory diseases such as diabetes (7–9), rheumatoid arthritis (RA) (10, 11), asthma (12, 13), fatty liver diseases (14, 15), and cancer (16–18). Mechanically, inflammation can induce changes in the epigenetic landscape in an inflammatory microenvironment (6, 19), and epigenetic modifications can in turn maintain and promote the development of inflammation by regulating the expression of various immune components (20, 21). With studies on the development of epigenetic modifications in inflammation and with rapid research progress on mechanisms and drug discovery, some star targets (lysine-specific demethylases (22, 23), BRD4 (16, 17, 24), EZH2-EED protein-protein interaction (25, 26), and HDACs (27)) have been used in the diagnosis or treatment of inflammatory diseases *in cellulo* and *in vivo*. Therefore, investigating the functions of epigenetic immune components in inflammatory diseases not only helps reveal the molecular mechanism of a variety of inflammatory diseases, but also develop novel theranostical strategies against these diseases.

This Volume II Research Topic continually collected excellent works on the “*Epigenetics of the Immune Component of Inflammation*,” and a total of 9 articles from 77 authors were accepted, which demonstrates the great interest in this Research Topic in this field, deepens the understanding of epigenetic regulation in immune diseases and inflammation responses, and highlights the clinical significance of epigenetic regulation and inflammatory immune components in disease theranostics. This Research Topic can be roughly divided into the following three subtopics.

## Genome modifications

Genome modifications mediate the progression of inflammatory diseases by modulating the expression of related inflammatory genes (28). In our Research Topic, Lagosz-Cwik et al. found that the DNA methyltransferase (DNMT) inhibitor decitabine could suppress the proliferation of gingival fibroblasts (GFs) and induce necrotic cell death via reducing genome methylation. RNA sequencing showed that decitabine raised chemokines CCL-5, -8, -13, and -20, IL-1A, -18, -33, CSF3, the matrix metalloproteinases MMP-1, -9, and -13, and intercellular adhesion molecule-1 (ICAM-1), and reduced genes mediated collagen fibril and extracellular matrix organization, which suggests that DNMT inhibitors are potential agents against periodontitis pathogenesis. However, the potential cytotoxicity of DNMT inhibitors is a non-negligible challenge for their clinical applications. Jiang et al. explored the methylation level of Homeodomain-interacting protein kinase 3 (HIPK3) in blood using a sample database including 235 RA patients, 30 osteoarthritis (OA) patients, and 30 matched healthy controls. The results revealed that all 7 CpG islands are hypomethylated in RA patients compared with OA and healthy individuals. The 33286785 CpG displays the highest predictive power (AUC=0.829) against RA, and the prediction model could be further improved by combining HIPK3 with clinical index rheumatoid factors (RF^+^) and anti-citrullinated protein antibodies (ACPA^−^). Moreover, the study also found that the methylated HIPK3 levels are negatively correlated with C-reactive protein (CRP), suggesting that the blood methylation level of HIPK3 holds potential as a clinical diagnostic biomarker and indicator for CRP in RA. Shan et al. summarized the latest application advancements of the gamma-aminobutyric acid (GABA)ergic system (mainly consisting of GABA, GABA transporter, and GABA-related receptors) in RA theranostics, which provides an insight into the potential theoretical guidance and clinical choices for RA therapy. Zheng et al. summed up the functions of serine protease granzymes (Gzms) in RA pathogenesis and showed that these enzymes are potential targets for diagnosis and therapy for RA. Natoli et al. found that the DNA methylation profiles in CD4^+^ T-cells could discriminate the disease status of healthy controls, skin psoriasis, and psoriatic arthritis, which suggests that DNA methylation imprints may be used to determine the degree and grade of psoriasis, and thus to help carry out individualized therapeutic strategies accordingly. Sapienza et al. revealed the distinction in the DNA methylation profiles of diabetes patients who eventually developed end-stage renal disease (ESRD) and without diabetic nephropathy (DN) (29) and Wang et al. showed that this profile could be used to discriminate diabetes with ESRD and without DN. Zhang et al. described the immunoregulatory and metabolic roles and action mechanisms of genome methylation modification in the progression of metabolic-associated fatty liver disease (MAFLD), which provides references for the diagnosis and treatment of MAFLD via targeting nucleotide methylation. Xue et al. found that 8-oxoguanine DNA glycosylase1 (OGG1) inhibition or ablation enhances the antiviral activity of epithelial cells toward infection of human respiratory syncytial virus (RSV) *in vitro* and *in vivo*. Further study revealed that OGG1 recognizes 8-oxoGua in the vicinity of interferon response elements (IRF) within the *IFN‐λ* promoter, and thus reduces the DNA occupancy of NF-κB/RelA and IRFs by promoting the interaction between the NF-κB homodimer p50 and p50 in guanine islets (5’-GGG-3’) in the *IFN‐λ* promoter, which reduces IFN‐λ production, increases viral load and neutrophilia, and finally aggravates viral infection and immunopathology in mice. This finding indicates that OGG1 is a potential target for eliminating pulmonary viral infections in clinical settings.

## Post-transcriptional modifications

Post-transcriptional modification refers to the various changes and alterations of RNA molecules after transcription from DNA (30). Post-transcriptional modifications including microRNA, lncRNA, tRNA, m^6^A modifications, etc. mediate various inflammatory diseases by modulating multiple immune components. Wang et al. systematically summarized the dysregulation of the miRNAs miR-183/96/182 cluster (miR-183C) in many autoimmune disorders, such as systemic multiple sclerosis, ocular autoimmune diseases, and lupus erythematosus, and highlighted the potential of miR-183C as targets for diagnosis markers and therapy against these autoimmune diseases. Wang et al. described the functions and mechanisms of non-coding RNAs including microRNAs, lncRNAs and m^6^A modifications in symptoms of podocytopathies, which provide a theoretical basis and target selection for the diagnosis and treatment of DN.

## Post-translational modifications

Post-translational modifications are also crucial epigenetic modes involved in accurately orchestrating a variety of inflammatory processes via writing, reading, and erasing marks of specific amino acid residues within proteins (31). Wang et al. showed that histone acetylation/de-acetylation modification in podocytopathy contributes to protecting from DN progression, which is a potential target for DN therapy.

## Author contributions

Y-JL: Investigation, Writing – original draft. H-JZ: Conceptualization, Resources, Validation, Writing – review & editing. HW: Conceptualization, Investigation, Resources, Validation, Writing – review & editing. C-MC: Conceptualization, Formal Analysis, Resources, Validation, Writing – review & editing. G-JY: Conceptualization, Supervision, Writing – original draft, Writing – review & editing.
